# Review on the Eyedrop Self-Instillation Techniques and Factors Affecting These Techniques in Glaucoma Patients

**DOI:** 10.1155/2016/9183272

**Published:** 2016-03-28

**Authors:** Ozcan Kayikcioglu, Sinan Bilgin, Murat Uyar

**Affiliations:** ^1^Department of Ophthalmology, Hafsa Sultan Hospital, Celal Bayar University, Faculty of Medicine, Manisa, Turkey; ^2^Department of Ophthalmology, Sifa University Faculty of Medicine, 35410 Izmir, Turkey

## Abstract

*Objective*. This study aims to evaluate eyedrop self-installation techniques and factors affecting these techniques in glaucoma patients.* Methods*. Researchers directly observed eyedrop instillation procedures of 66 glaucoma patients. Contact with periocular tissues and instillation onto ocular surface or conjunctival fornices were considered. Correlations of instillation patterns with patient characteristics including age, gender, intraocular pressure, cup-to-disc ratio, visual field loss, and total intake of glaucoma medication and handgrip strength score were searched.* Results*. The average handgrip strength in the instillation without periocular contact group was 66.4 ± 19.7 kg, while the average handgrip strength score was 55.9 ± 20.9 kg in the instillation with contact group. The difference between the two groups was statistically significant (*p* = 0.039). No statistically significant correlation was found between handgrip strength and the mean number of glaucoma medications, c/d, intraocular pressure (*p* > 0.05). Also there was no significant relation between mean handgrip strength score and the severity of the visual field defect (*p* = 0.191).* Conclusion*. Patients especially with severe glaucomatous damage should be adequately instructed about the proper techniques for self-instillation of eyedrops and motivated to use a proper technique. Also, it is possible to suggest that patients with a higher handgrip strength, indicating the well-being of general health, may be doing better in properly instilling glaucoma eyedrops.

## 1. Introduction

Proper administration of medicines is as important as the proper choice of medicines for a successful treatment, since, whatever the effectiveness of the medicine is, the benefit from the treatment depends on the correct administration. In glaucoma patients, poor adherence and compliance with medical treatment were found to be associated with difficulty in achieving target intraocular pressure (IOP) and larger visual field defects have been reported in this group of patients [1–4].

Adherence to treatment is a complex issue having many aspects related to the patient, such as accepting the disease, continuing to have repeat prescriptions, and applying the right daily medication regime at suggested intervals. One important aspect for glaucoma patients is difficulties during the administration of their eyedrops. Missing the eye during the administration of eyedrops, wasting extra drops to achieve a successful eyedrop instillation, and contamination of eyedrop bottles during eyedrop instillation are among these difficulties [5].

Aging is associated with reduced daily living activities as a result of health problems along with reduced social and functional capacities. Handgrip strength is critical for being able to perform a number of daily living activities [6]. Although handgrip strength is generally used as an indicator of overall physical strength and health, it is also accepted as an indicator of functional limitation and disability [7, 8]. Hand dynamometers have been developed to measure handgrip strength, reflecting the general functional level of the individual. Jamar hand dynamometer is a validated, reliable instrument recommended by The American Society of Hand Therapists (ASHT) as a gold standard to measure handgrip strength [9].

To the best of our knowledge, this is the first time that a group was analysed to examine whether handgrip strength is an independent variable in the self-administration success of eyedrops.

## 2. Materials and Methods

Initially 70 patients with primary open angle glaucoma (POAG) were included in this prospective, consecutive, and observational study. Four patients were excluded because of missing data and visual fields, so 66 patients were analysed. The study was carried out with approval from the local institutional review board and adhered to the tenets of the Declaration of Helsinki. Informed consent was obtained from all participants.

### 2.1. Eyedrop Instillation Technique

Subjects were uniformly instructed to instill the artificial tears just as they would instill their medications at home. All subjects instilled drops from a sterile 5 mL bottle of sterile artificial tears. Afterwards, the patients' eyedrop instillation technique was recorded by the observer.

Patients were asked to hold the dynamometer in their dominant hand and squeeze it with maximum strength. Three measurements were recorded with one-minute intervals. The results were in weight (kilogram). Three measurements of strength were recorded between 8:00 a.m. and 10:00 a.m. while the patient was in the seated position. In this study, the Jamar hand dynamometer (Sammons Preston Inc., Bolingbrook, IL) was used to calculate the mean handgrip strength score of the patients.

SITA strategies (24-2 or 10-2) were used from the Humphrey Field Analyser II (HFA; Model 750, Zeiss-Humphrey, Inc., Dublin, CA). If there was no visual field within the previous 6 months the patient underwent a visual field test. For visual field tests, the reliability parameters of less than 20% errors were used. Visual field defects were classified as mild (Mean Deviation (MD) ≥ −6 dB), moderate (−6 dB > MD > −12 dB), and severe (MD ≤ −12 dB), based on the Hodapp-Parrish-Anderson criteria [10].

The inclusion criteria were primary open angle glaucoma patients who had the ability to administer eyedrops by themselves and having topical antiglaucomatous medication for more than one year.

The exclusion criteria were diseases that could cause tremor such as Parkinson's disease and Parkinson-like syndromes, dementia that could impact on the reliability of patient self-reports, and muscle and joint disorders that could lead to a limited range of motion. Patients whose VA were less than 0.4 logMAR were not included in the study. Low visual acuity might influence eyedrop instillation capability.

The patients' age, gender, intraocular pressure, cup-to-disc ratio, visual field loss, and total intake of glaucoma medication were also recorded.

### 2.2. Statistical Analysis

Statistical analyses were performed in the R programming language using Rstudio version 0.98.501 software. The conformity of the variables with a normal distribution was analysed using analytic methods (Kolmogorov-Smirnov/Shapiro-Wilk tests). The mean ± standard deviation was used in the descriptive statistics of the normally distributed data. Pearson's correlation analysis was used to find the correlation between the discrete variables. *t*-test was used to analyse the comparison of normally distributed continuous variables between the independent groups. Chi-square test was used to compare baseline characteristics between the groups. A *p* value less than 0.05 was considered as statistically significant.

## 3. Results

**Figure 1:**
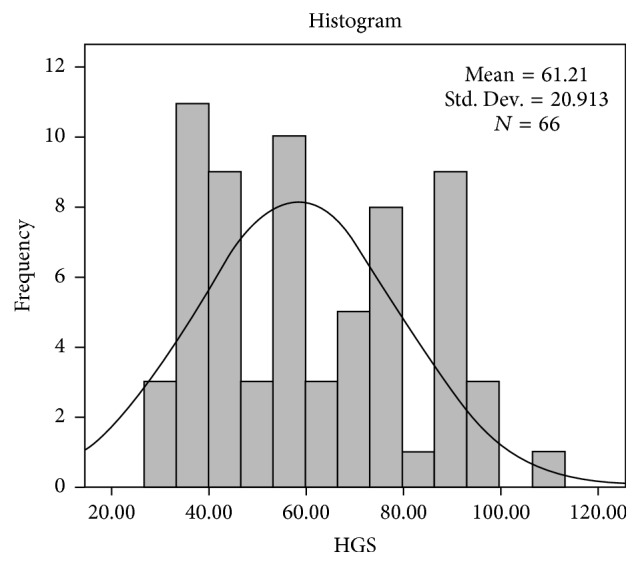
Frequency of handgrip stength (HGS) scores.

Patients were divided into six subgroups according to the self-instillation techniques. Some of the patients were using more than one of these techniques. For example, while patients instillated eyedrops in the seated position, they were bringing the tip of the dropper into contact with the globe or periocular tissue. Patient's characteristics and eyedrop instillation techniques are shown in Tables 1 and 2, respectively. Also, frequency of handgrip stength (HGS) scores is shown in Figure 1.

No statistically significant difference was found between the patients instilling eyedrop in seated position and supine position in age, gender, handgrip strength, the mean number of glaucoma medications, visual field defects, c/d, and intraocular pressure (*p* > 0.05). However, visual acuity was higher in the seated position group compared to the supine position group (*p* = 0.005) (Table 3).

In the comparison of the patients who brought the dropper tip into contact with any part of the globe or eyelids and the patients who did not, the average handgrip strength score in the instillation without contact group was 66.4 ± 19.7 kg, while it was 55.9 ± 20.9 kg in the instillation with contact group. The difference between the two groups was statistically significant (*p* = 0.039). However, no significant difference was found between these two groups in regard to age, gender, intraocular pressure, the mean number of glaucoma medications, and visual field defects (*p* > 0.05) (Table 3).

No significant difference was found between the group of patients who instilled their eyedrop into the inner canthal region and into the inferior conjunctival cul-de-sac, in age, gender, handgrip strength, the number of glaucoma medications, and c/d (*p* > 0.05). However, a statistically significant difference was found between those two groups, in intraocular pressure and visual field defect (*p* = 0.037, *p* = 0.043, resp.) (Table 3).

No statistically significant correlation was found between handgrip strength score and the mean number of glaucoma medications, c/d, intraocular pressure (*p* > 0.05). Also there was no significant relation between mean handgrip strength score and the severity of the visual field defect (*p* = 0.191).

## 4. Discussion

The overall success of the glaucoma treatment depends on the patient compliance with each individual step included in the process of treatment. Sleath et al. reported that the topical administration of ocular medication was the most challenging part of the therapy [11]. In clinical trials, the rate of any contact between the tip of the eyedrop bottle and the eye and/or the eyelid has been reported as 21.9% to 80% [5, 12–15]. These rates are closely related to the rates of bottle contamination, corneal ulceration, and financial issues related to wasted medicines [5, 16, 17].

Individuals with high handgrip strength value are suggested to perform many daily activities which require finger and hand coordination, such as holding knives, forks, and spoons. So we hypothesize that eyedrop instillation is probably affected by handgrip strength. In this study, patients having higher handgrip strength showed a tendency to instill their eyedrop without bringing the tip of the bottle into contact with the eye or eyelids. However, no significant relationship was found between the handgrip strength which is used as an indicator of the functional limitations and disabilities and glaucoma parameters including IOP and visual field defect.

Although any significant effect of higher handgrip strength on glaucoma was not demonstrated in patients able to self-administer their eyedrop, this evidence suggested that higher handgrip strength may be associated with proper self-instillation of eyedrops and a lower risk of contamination. Handgrip strength depends on age, stress, nutrition, and general medical state of the subject [7, 18, 19]. Therefore, in addition to prescriptions, recommendations aimed at improving their lifestyle and general medical condition may also be important in the medical care of glaucoma patients.

In some studies, no significant relationship was found between the proper self-instillation of the eyedrop and the severity of visual field defect and visual acuity [2, 12].

In the current study, significant differences were found between the group of patients who instilled their eyedrop into the inner canthal region and those who instilled their eyedrop into the inferior conjunctival cul-de-sac, in visual field defect severity and IOP measurements. As expected, the visual field defect severity and IOP were lower in the inferior conjunctival cul-de-sac group compared to the inner canthal region group. Therefore, patients especially with severe glaucomatous damage should be adequately instructed about the proper techniques for self-instillation of eyedrops and motivated to use a proper technique.

In a study in glaucoma patients, Hennessy et al. evaluated the relationships between the proper eyedrop technique and age and gender [20]. No relationship was found between the proper eyedrop technique and sex, while patients under the age of 70 years were particularly successful in self-instillation of eyedrops. Stone et al. reported more successful eyedrop techniques among the male patients and patients with better visual functions [15]. Similarly, we did not found significant relationship between the eyedrop techniques and age and gender.

Limitation of this study was the lack of the assessment of the relationship between the preference of eyedrop technique and educational level and socioeconomic status of the patients. Also we used only one visual field to classify patients, and follow-up data was not included which would give important information about treatment success. Additionally, small population size and not using videotaped method for observation of eyedrop instillation techniques were other limitations of this study.

## 5. Conclusions

Even though this study has limitations, patients especially with severe glaucomatous damage should be adequately instructed about the proper techniques for self-instillation of eyedrops and motivated to use a proper technique. Also, although this subject requires further studies, it is possible to suggest that patients with a higher handgrip strength, indicating the well-being of general health, may be doing better in successful instillation of eyedrops.

## Figures and Tables

**Table 1:** Patients' characteristics (*N* = 66).

Variable	
Age (years)	61.69 ± 9.6 (38–82)
Gender% (*n*)	
Female	54.5% (36)
HGS (kilogram)	61.2 ± 20.9
Visual field defect	
Mild	54.5% (36)
Moderate	12.1% (8)
Severe	33.3% (22)
BCVA (logMAR)	0.15 ± 0.52
c/d	0.5 ± 0.2
Glaucoma medications% (*n*)	
1	59.1% (39)
2	21.2% (14)
3	19.7% (13)

HGS, handgrip strength; BCVA, best-corrected visual acuity; logMAR, logarithm of the minimal angle of resolution; c/d, cup-to-disc ratio.

**Table 2:** Patients' eyedrop instillation techniques (*N* = 66).

Instillation technique	% (*n*)
Seated position (Group 1)	50% (33)

Supine position (Group 2)	50% (33)

Self-instillation without bringing the tip of the dropper (Group 3) into contact with the globe or periocular tissue	50% (33)

Bringing the tip of the dropper into contact with (Group 4) the globe or periocular tissue while instilling eyedrops	50% (33)

Self-instillation into the (Group 5) inferior conjunctival cul-de-sac	65% (43)

Self-instillation into the (Group 6) inner canthal region	35% (23)

**Table 3:** The relationships between the eyedrop techniques and age, handgrip strength, the number of active pharmaceutical ingredients, intraocular pressure, visual acuity, and cup/disc ratio (*N* = 66).

Variable	Group 1 (*n* = 33)	Group 2 (*n* = 33)	Group 3 (*n* = 33)	Group 4 (*n* = 33)	Group 5 (*n* = 43)	Group 6 (*n* = 23)
Handgrip strength	64.4 ± 20.6	57.9 ± 21	66.4 ± 19.7	55.9 ± 20.9	62.4 ± 20.7	56.2 ± 21.8
	*p* = 0.211		***p* =**0.039^*∗*^		*p* = 0.347	

Visual field defect% (*n*)						
Mild	42.4% (14)	66.7% (22)	54.5% (18)	54.5% (18)	65.1% (28)	34.7% (8)
Moderate	12.1% (4)	12.1% (4)	12.1% (4)	12.1% (4)	06.9% (3)	21.7% (5)
Severe	45.5% (15)	21.2% (7)	33.3% (11)	33.3% (11)	27.9% (12)	43.4% (10)
	*p* = 0.096		*p* = 1		***p* =**0.043^*∗*^	

BCVA (logMAR)	0.1–0.7	0.3–0.52	0.15–0.52	0.22–0.52	0.15–0.52	0.3–0.52
	***p* =**0.005^*∗*^		*p* = 0.558		*p* = 0.067	

Intraocular pressure	18.7 ± 4.8	21.9 ± 18.7	21 ± 8.2	19.5 ± 5.4	19.4 ± 4.9	23.9 ± 11.8
	*p* = 0.064		*p* = 0.392		***p* =**0.037^*∗*^	

Glaucoma medications	1.5 ± 0.7	1.7 ± 0.8	1.5 ± 0.7	1.6 ± 0.8	1.5 ± 0.7	1.6 ± 0.8
	*p* = 0.361		*p* = 0.543		*p* = 0.668	

c/d	0.5 ± 0.1	0.6 ± 0.2	0.5 ± 0.2	0.5 ± 0.2	0.6 ± 0.2	0.5 ± 0.2
	*p* = 0.083		*p* = 0.958		*p* = 0.165	

BCVA, best-corrected visual acuity; logMAR, logarithm of the minimal angle of resolution; c/d, cup-to-disc ratio.

^*∗*^Statistically significant differences.

## References

[1] Konstas A. G. P., Maskaleris G., Gratsonidis S., Sardelli C. (2000). Compliance and viewpoint of glaucoma patients in Greece. *Eye*.

[2] Sleath B., Blalock S., Covert D. (2011). The relationship between glaucoma medication adherence, eye drop technique, and visual field defect severity. *Ophthalmology*.

[3] Nordmann J.-P., Baudouin C., Renard J.-P. (2010). Measurement of treatment compliance using a medical device for glaucoma patients associated with intraocular pressure control: a survey. *Clinical Ophthalmology*.

[4] Friedman D. S., Okeke C. O., Jampel H. D. (2009). Risk factors for poor adherence to eyedrops in electronically monitored patients with glaucoma. *Ophthalmology*.

[5] Gupta R., Patil B., Shah B. M., Bali S. J., Mishra S. K., Dada T. (2012). Evaluating eye drop instillation technique in glaucoma patients. *Journal of Glaucoma*.

[6] Nicolay C. W., Walker A. L. (2005). Grip strength and endurance: influences of anthropometric variation, hand dominance, and gender. *International Journal of Industrial Ergonomics*.

[7] Massy-Westropp N., Rankin W., Ahern M., Krishnan J., Hearn T. C. (2004). Measuring grip strength in normal adults: reference ranges and a comparison of electronic and hydraulic instruments. *Journal of Hand Surgery*.

[8] Rantanen T., Guralnik J. M., Foley D. (1999). Midlife hand grip strength as a predictor of old age disability. *The Journal of the American Medical Association*.

[9] Roberts H. C., Denison H. J., Martin H. J. (2011). A review of the measurement of grip strength in clinical and epidemiological studies: towards a standardised approach. *Age and Ageing*.

[10] Hodapp E., Parrish R. K., Anderson D. R. (1993). *Clinical Decisions in Glaucoma*.

[11] Sleath B., Robin A. L., Covert D., Byrd J. E., Tudor G., Svarstad B. (2006). Patient-reported behavior and problems in using glaucoma medications. *Ophthalmology*.

[12] Hennessy A. L., Katz J., Covert D., Protzko C., Robin A. L. (2010). Videotaped evaluation of eyedrop instillation in glaucoma patients with visual impairment or moderate to severe visual field loss. *Ophthalmology*.

[13] Dietlein T. S., Jordan J. F., Lüke C., Schild A., Dinslage S., Krieglstein G. K. (2008). Self-application of single-use eyedrop containers in an elderly population: comparisons with standard eyedrop bottle and with younger patients. *Acta Ophthalmologica*.

[14] Sleath B., Blalock S. J., Stone J. L. (2012). Validation of a short version of the glaucoma medication self-efficacy questionnaire. *British Journal of Ophthalmology*.

[15] Stone J. L., Robin A. L., Novack G. D., Covert D. W., Cagle G. D. (2009). An objective evaluation of eyedrop instillation in patients with glaucoma. *Archives of Ophthalmology*.

[16] Solomon A., Chowers I., Raiskup F., Siganos C. S., Frucht-Pery J. (2003). Inadvertent conjunctival trauma related to contact with drug container tips: a masquerade syndrome. *Ophthalmology*.

[17] Geyer O., Bottone E. J., Podos S. M., Schumer R. A., Asbell P. A. (1995). Microbial contamination of medications used to treat glaucoma. *British Journal of Ophthalmology*.

[18] Poornima K. N., Karthick N., Sitalakshmi R. (2014). Study of the effect of stress on skeletal muscle function in geriatrics. *Journal of Clinical and Diagnostic Research*.

[19] Norman K., Stobäus N., Gonzalez M. C., Schulzke J.-D., Pirlich M. (2011). Hand grip strength: outcome predictor and marker of nutritional status. *Clinical Nutrition*.

[20] Hennessy A. L., Katz J., Covert D. (2011). A video study of drop instillation in both glaucoma and retina patients with visual impairment. *American Journal of Ophthalmology*.

